# ‘Conga lines’ of Ediacaran fronds: insights into the reproductive biology of early metazoans

**DOI:** 10.1098/rsos.231601

**Published:** 2024-05-29

**Authors:** Katie M. Delahooke, Alexander G. Liu, Nile P. Stephenson, Emily G. Mitchell

**Affiliations:** ^1^ Department of Earth Sciences, University of Cambridge, Cambridge, UK; ^2^ Department of Zoology, University of Cambridge, Cambridge, UK; ^3^ Museum of Zoology, University of Cambridge, Cambridge, UK

**Keywords:** Ediacaran, rangeomorphs, reproduction, plasticity, stolons, CFD

## Abstract

Late Ediacaran strata from Newfoundland, Canada (~574–560 Ma) document near-census palaeocommunities of some of the earliest metazoans. Such preservation enables reproductive strategies to be inferred from the spatial distribution of populations of fossilized benthic organisms, previously revealing the existence of both propagule and stoloniferous reproductive modes among Ediacaran frondose taxa. Here, we describe ‘conga lines’: linear arrangements of more than three closely spaced fossil specimens. We calculate probabilistic models of point maps of 13 fossil-bearing bedding surfaces and show that four surfaces contain conga lines that are not the result of chance alignments. We then test whether these features could result from passive pelagic propagules settling in the lee of an existing frond, using computational fluid dynamics and discrete phase modelling. Under Ediacaran palaeoenvironmental conditions, preferential leeside settlement at the spatial scale of the conga lines is unlikely. We therefore conclude that these features are novel and do not reflect previously described reproductive strategies employed by Ediacaran organisms, suggesting the use of mixed reproductive strategies in the earliest animals. Such strategies enabled Ediacaran frondose taxa to act as reproductive generalists and may be an important facet of early metazoan evolution.

## 1. Introduction

Metazoans exhibit a remarkable range of reproductive strategies, each of which has varied costs and benefits [1]. Trade-offs between these costs and benefits can result in mixed reproductive strategies, as exhibited by extant members of some of the earliest diverging metazoan phyla, including poriferans and cnidarians [1,2]. Mixed reproductive strategies may manifest in a number of ways. For example, a single organism may employ multiple reproductive modes either simultaneously, or throughout its lifespan [3,4]. Alternatively (and often in addition), reproductive mode may be controlled by phenotypic plasticity [1,4]: the production of varied phenotypes from a genotype in response to different environmental cues. The abundant presence of mixed reproductive strategies in the earliest diverging phyla may suggest that the first animals also exhibited mixed reproductive strategies.

Macroscopic metazoans first appear in the rock record in the Ediacaran strata (~580–538 Ma [5]). Some of the earliest metazoan taxa, within the Avalon assemblage, are preserved in their thousands on extensive bedding planes in Newfoundland (Canada), and the United Kingdom [6–10] and provide opportunities to investigate reproductive dynamics in early animal ecosystems (e.g. [11]). The sessile, soft-bodied Avalonian macro-organisms are preserved *in situ* as casts and moulds [6], often beneath volcaniclastic sediments, in turbiditic sequences deposited within deep-marine palaeoenvironments [12–14]. The Avalon assemblage precedes the advent of widespread mobility, biomineralization and predation present in later Ediacaran assemblages [6,8], and is instead dominated by sessile soft-bodied rangeomorphs and arboreomorphs: enigmatic frondose organisms seemingly restricted to the late Ediacaran Period [15,16] (although morphologically similar forms are rarely described from the early Cambrian [17,18]). The rangeomorphs and arboreomorphs are considered to have stem- and total-group eumetazoan affinities, respectively [19,20], and existed alongside examples of total- and crown-group Cnidaria [21,22] and candidate sponges [23,24] in diverse benthic palaeocommunities [25,26].

**Figure 1.**
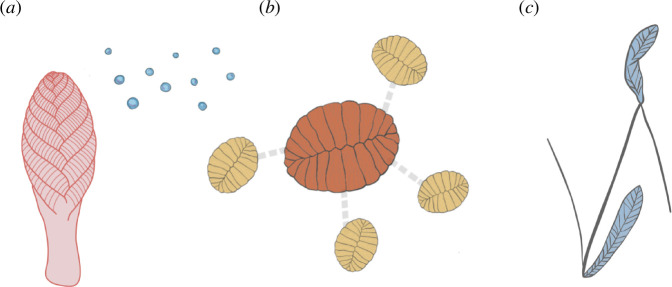
Schematic reconstructions of previously described rangeomorph reproductive methods. (*a*) Propagules in lateral view [10,25,27]. (*b*) Stolons forming small isotropic clusters in plan view [10]. (*c*) Stolons reaching over 1 m in length, sometimes connecting specimens, in plan view [28].

Rangeomorphs have been suggested to be capable of reproducing by stolons, fragments, buds and pelagic propagules (figure 1) on the basis of spatial distributions across multiple scales, as well as direct evidence from fossil impressions [11,26–30]. Different reproductive strategies manifest as different spatial patterns of sessile organisms. The use of methods such as spatial point pattern analysis (SPPA) can disentangle reproductive processes from environmental and interactive influences. Such analyses have been used to describe the patterns produced by known reproductive processes in extant marine sessile invertebrates (e.g. [31,32]). The near census preservation of Avalonian palaeocommunities permits the application of these techniques to elucidate rangeomorph reproductive biology [11,25,26,33,34]. For example, the rangeomorph *Fractofusus* has been statistically demonstrated to form small, isotropic (i.e. non-directional) and hierarchical clusters, comprising smaller specimens clustered around larger specimens, which were inferred to arise from stoloniferous reproduction [11].

More recently, observed filamentous connections between rangeomorphs were interpreted to be stoloniferous, on the basis of the filaments terminating at the bases of individual specimens, and their large size (sometimes extending over a metre) being incompatible with fungal or bacterial affinities [27]. Evidence for pelagic propagules derives from the wide palaeogeographic distribution of taxa such as *Charnia* [26,28], and current-influenced spatial distributions [11], but the nature of the propagules remains unresolved. Larvae, gametes, embryos and microscopic buds or fragments are all possible forms of pelagic propagules given existing data and rangeomorph phylogenetic placement [19,35]. Putative macroscopic buds and fragments have been suggested to occur in *Culmofrons* and *Avalofractus* [29,30], although the approximately fractal branching [36] of rangeomorphs may make it hard to distinguish such fragments from whole organisms (e.g. [29]).

### 1.1. ‘Conga lines’ and explanations for aligned specimens

**Figure 2.**
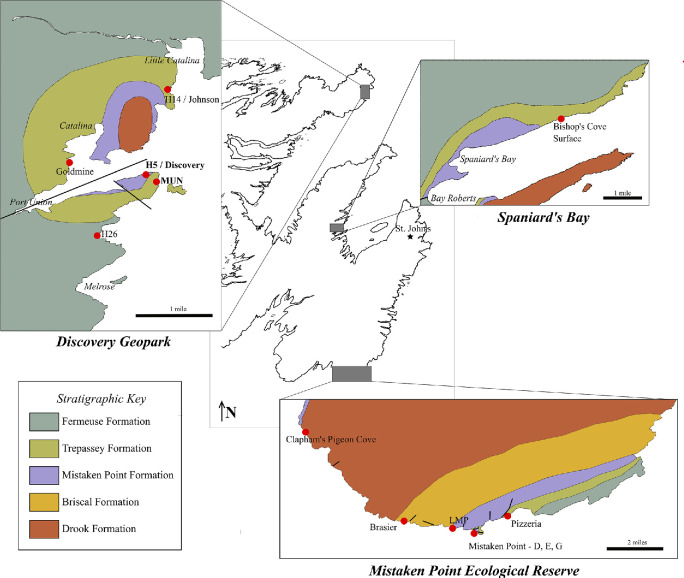
Map of studied fossil-bearing surfaces in southeast Newfoundland, Canada. Geology and stratigraphy follow: Spaniards Bay [13]; Discovery Geopark [37]; Mistaken Point [9]. Italic text indicates local towns. Bold black lines indicate geological faults.

**Figure 3.**
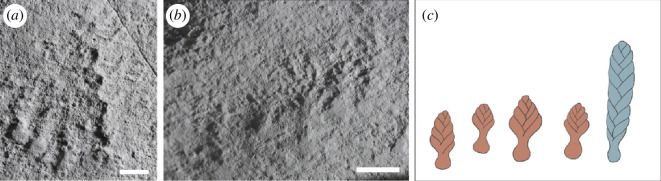
‘Conga lines’ of rangeomorph fronds on the H5 surface, Discovery Geopark, Newfoundland, Canada, with larger frond on the right. (*a, b*) Scale bars = 1 cm. (*c*) Schematic illustration of specimens in (*b*), where the blue frond is larger than the orange fronds.

On the H5 fossil-bearing bedding plane in the Discovery Geopark, Newfoundland, Canada (figure 2), we observed examples of frondose specimens forming closely spaced, linear arrangements of 3–6 specimens, which are here termed ‘conga lines’ (figure 3). In two observed cases, conga lines appear to have a frond at one end of the line that is substantially larger than the others (figure 3). Such aligned arrangements of specimens have not previously been reported from Ediacaran fossil assemblages.

The spatial arrangement of sessile marine invertebrates reflects the interplay between abiotic factors, reproductive processes and interactions such as competition [38]. Linear arrangements of sessile organisms, such as those that form the conga lines, could only feasibly arise from a much narrower set of such factors. First, they could result from random chance, particularly if specimens occur in high densities or are aggregated due to other processes, due to the increased probability that they will seemingly align. Second, the interaction of a directional abiotic factor, such as a current, with reproductive processes could result in a linear pattern of recruitment, which would be influenced by the nature of the reproductive propagules. We consider two end members of reproductive propagules based on their dispersal capacity: pelagic and philopatric (*sensu* [39]). Here, pelagic is used to describe passive propagules that are neutrally or positively buoyant, and those capable of active swimming, with long-distance (teleplanic or anchiplanic) dispersal capacity. Pelagic propagules may include larvae, gametes, embryos, and microscopic buds or fragments, with larvae typically developing planktonically or demersally [40]. Conversely, philopatric (remaining within centimetres to a couple of metres of their parent) propagules are negatively buoyant (e.g. [41]) or capable of active swimming, have an aplanic dispersal capacity (immediate settling), and may demonstrate benthic (including parental) development [40,42]. Philopatric propagules include macroscopic buds or fragments, some embryos and larvae (often brooded or crawling [40,43]). Flow patterns around an obstacle may lead to increased settling of passive pelagic propagules in a linear fashion, or alternatively, the sequential production of philopatric propagules may interact with a current to create linear features in the lee of the parent organism. Third, linear arrangements of sessile organisms may be purely biological, e.g. a consequence of a non-branching, reproductive runner-like stolon connecting multiple organisms in sequence.

In this study, we seek to determine whether the newly documented Ediacaran conga lines are the consequence of chance alignments by calculating probabilistic models of point maps of 13 fossil-bearing surfaces from Newfoundland, Canada. We then test whether conga lines could be formed from passive pelagic propagule settlement in the lee of an obstacle, using computational fluid dynamics and particle tracking. Following these tests, we then consider the most likely mode of formation of the conga lines and the consequences for early metazoan evolution.

## 2. Methods

### 2.1. Data collection

We analysed point maps of portions of 13 late Ediacaran fossiliferous surfaces from Newfoundland, Canada, comprising two surfaces newly mapped in this study (H5 and MUN from the Discovery Geopark), supplemented by 11 further surfaces compiled by [44] incorporating data from [25] (Bishop’s Cove in the Spaniard’s Bay area; Goldmine, H14 and H26 in the Discovery Geopark; Brasier (also known as BR5), Clapham’s Pigeon Cove (CPC), ‘D’ Surface, ‘E’ Surface, ‘G’ Surface, Pizzeria and Lower Mistaken Point (LMP) in Mistaken Point Ecological Reserve; figure 2). Together, these point maps document 16 002 specimens over 425 m^2^ (electronic supplementary material, table S1). This dataset excluded available published data from surfaces that are unsuitable for spatial analyses due to tectonic deformation (Shingle Head), non-typical sedimentology (UIC), small areal extent (Bristy Cove) or ambiguity surrounding the contemporary nature of discoidal fossils (Bed B).

Well-preserved areas of the H5 and MUN surfaces [37,45] were moulded in the field using silicone rubber. A total of eight moulds encompassing 4.11 m^2^ were created for H5, and five moulds encompassing 3.91 m^2^ were created for MUN. Jesmonite casts were subsequently made from these moulds (housed in the Sedgwick Museum of Earth Sciences, Cambridge, UK; H5: CAMSM X 50342.1–9, MUN: CAMSM X 50340.1–6). Photogrammetry of casts under controlled lighting conditions was used to produce high-resolution two-dimensional orthomosaics, which were then scaled and aligned using photogrammetric maps of the entire surface taken in the field. The orthomosaics were subsequently checked against LiDAR scans for accuracy (using Agisoft Metashape Professional 1.8.4., cf. [25]). Vector maps were then produced from these orthomosaics in Inkscape 1.0.1, documenting specimen spatial position, dimensions (frond length, frond width, stem length and stem width), orientation and taxonomic identification (cf. [25]), and processed using a custom R script (https://github.com/kmdelahooke/dex) to generate point maps of the two surfaces (electronic supplementary material, figures S1 and S2). To correct for tectonic deformation, the point coordinates were retrodeformed by returning ovate discoidal fossils to circles (cf. [11,12]). To investigate the influence of retrodeformation and the presence of large cracks, small holes and eroded patches on our analyses, additional point maps of the H5 surface were created for retrodeformed data, non-retrodeformed data, and a case where all small holes and eroded areas of the surface were masked (i.e. removed), to assess the sensitivity of our methods to small-scale, patchy erosion.

### 2.2. Probabilistic modelling

To investigate whether the conga lines result from chance alignments, we used both analytical calculations and numerical probabilistic modelling. First, we calculated the probability of finding the observed number of conga lines on each surface analytically (i.e. by calculating the exact solution using derived equations, §2.2.3). Second, we verified the analytical calculations using numerical Monte–Carlo simulations of the H5 surface (§2.2.4). Probability analyses were performed using custom code in R 4.2.2 (R Core Team 2022), using the *spatstat* package [46]. All codes are available on Github: https://github.com/kmdelahooke/Conga-Lines.

#### 2.2.1. Spatial point process analysis

**Figure 4.**
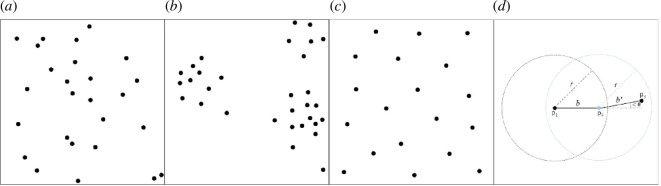
Schematic examples of different types of point processes. (*a*) Complete spatial randomness (CSR), modelled by a homogenous Poisson process. (*b*) Aggregation, here modelled by a Thomas cluster process. (*c*) Segregation, here modelled by a Gibbs process. (*d*) Visual representation of the method used to calculate alignments of three points (*p_1_, p_2_, p_3_*), where r is the maximum spacing between points, θ is the maximum angle between points and b is the bearing between those points, such that | *b*′ − *b* |< θ.

The spatial distribution of points (specimens) on each surface was characterized prior to probabilistic calculations, as the background distribution of points naturally affects the likelihood that chance alignments will be found. At the simplest level, points can be randomly distributed, aggregated (points closer together than random) or segregated (points further apart than random; figure 4*a*–*c*). Intuitively, if the density of points for any of these patterns increases, the probability of chance alignments also increases. If the pattern is aggregated, it would be more likely that one point is proximal to another, but the inverse is true if points are highly segregated. Second-order summary functions, such as the K-function or pair correlation function (PCF), can be used to quantify these patterns (aggregation, segregation or a random distribution) across a range of spatial scales [47].

Point process models are statistical models that generate point patterns within an observation window. In order to allow the point pattern to be characterized by a series of parameters, these models can be fitted to a summary function of a given point pattern, such as density (first-order intensity function) or the K-function or PCF (second-order functions [38,47]). Most simply, the points could be randomly distributed: complete spatial randomness (CSR, figure 4*a*) is modelled by a homogeneous Poisson process parameterized by density (* λ *), equivalent to the number of points ( n ) (here specimens) over a given area ( A ) (here mapped area). Alternatively, points could be aggregated, which can be modelled by processes including a Thomas cluster process that can describe reproductive aggregations (figure 4*b*), or heterogeneous Poisson processes, which best describe aggregation due to shared habitat associations [47]. Thomas cluster processes are parameterized by the density of parent points ( κ ) (themselves following a homogenous Poisson distribution), the mean number of offspring points in the cluster ( μ ) and the standard deviation of the distance of the offspring points to their parent point ( σ ). Conversely, points could be segregated, which can be modelled by a suite of processes including the Gibbs process (figure 4*c*). Many Avalonian populations show clustered distributions, of which Thomas cluster (TC) and double Thomas cluster (DTC) processes dominate [25].

#### 2.2.2. Background distribution model fitting and evaluation

Both homogenous Poisson and Thomas cluster processes were fitted to point patterns for each surface using the package *spatstat* [46] as follows:

1. Homogeneous Poisson process (CSR) models were fitted to the density (intensity) function of the point pattern using the maximum likelihood method [48], generating parameter λ .
2. Single TC models were fitted to the PCF of the point pattern using the minimum contrast method, generating parameters κ,μ,σ [49].
3. The best process to describe a point pattern was determined using Diggle’s goodness-of-fit test [47,50].
4. The point process model with the highest goodness-of-fit (*p*_d_) value was used as the background distribution when calculating the probability of chance alignments (e.g. [25,47]).

#### 2.2.3. Predicted number of congas—analytical method

The predicted number of chance alignments was determined analytically assuming both CSR and Thomas clustered background distributions. The predicted number of chance three-point alignments, of maximum spacing r mm and maximum angle θ °, was derived from Ripley’s K-function (electronic supplementary material, §2.1), which describes the likelihood of finding another point within a certain radius of a given point of the point pattern [51].

Given a CSR background distribution, where λ is the density of points and A is the mapped area, the predicted number of three-point alignments on a surface ( $E_{pois}$ ) is,


(2.1) $$E_{pois}=\frac{\theta \pi^{2}r^{4}\lambda^{3}A}{360}.$$

Using a Thomas clustered distribution, where μ,κ,σ are the fitted TC parameters (see §2.2.1), and A is the mapped area, the predicted number of three-point alignments on a surface ( $E_{TC}$ ) is,


(2.2) $$E_{TC}=\frac{\theta A\kappa^{3}\mu^{3}}{360}{\left(\pi r^{2}+\frac{1-exp\left(-\frac{r^{2}}{4\sigma^{2}}\right)}{\kappa }\right)}^{2}.$$

#### 2.2.4. Predicted number of congas—numerical method

The analytic models were validated numerically on the H5 surface as follows:

1. CSR and TC models were fitted, as outlined in §2.2.2 (steps 1 and 2), to the entire surface point pattern.
2. Ten thousand Monte–Carlo simulations of both random and clustered point processes were generated using *spatstat*, using the parameters from fitted homogeneous Poisson (* λ* = 140 m^−2^) and TC models ( κ = 18 m^−2^, μ = 6, σ = 72.63 mm), and within a window defined by the H5 surface outline.
3. For each simulation, we ran an algorithm (https://github.com/kmdelahooke/Conga-Lines) to detect linear features consisting of at least three aligned points (parameterized by r and θ ), and the number of three-point alignments found at that iteration was recorded. This algorithm visits each point in the point pattern (*p_1_*) and finds any points that are within a radius *r* mm (*p_2_*). For each of these points, any points within *r* mm (*p_3_*) are detected (figure 4*d*). All prior points in the sequence are excluded from the search. The bearing (angle) between *p*_1_ and *p*_2_ (*b*) as well as *p*_2_ and *p*_3_ (*b′*) is calculated (figure 4*d*). If the bearings are within a certain angle *θ*, such that | *b′ − b |< θ,* then the coordinates of each point were recorded. The number of alignments found for each simulated point pattern was returned.
4. Step 3 was repeated, while holding all other parameters at a control on the surface (electronic supplementary material, tables S2 and S3), for the following range of values:
  -
r : 10, 12, 15 and 20 mm (control: 15 mm) encompassing the variation in observed spacing of conga lines on the H5 surface.
  -
θ : 20°, 25°, 30° and 45° (control: 30°), encompassing strong to weak alignments.
  - CSR density parameter: 10, 50, 100 and 150 specimens m^−2^ (control: 143 specimens m^−2^), encompassing typical fossil densities of Avalonian macrofossil-bearing surfaces.
  - TC parameters, encompassing typical parameters found for Avalonian macrofossil-bearing surfaces (e.g. [11]):
    -
κ : 10, 75, 150, 200 × 10^6^ m^−2^ (control: 55 × 10^6^ m^−2^).
    -
μ : 2, 3, 4, 6 (control: 3).
    -
σ : 20, 45, 75, 120 mm (control: 45 mm).

The relationship between the predicted number of three-point alignments and each parameter could then be compared between both analytical and numerical methods (see §2.2.6).

#### 2.2.5. Probability distribution

A probability distribution for each set of parameters was drawn from the Monte–Carlo simulations by plotting the number of times a simulation returned a given number of three-point alignments. This probability distribution follows a Poisson distribution, $P\left(x\right)=\frac{e^{-E_{D}\;\;}E_{D}^{x}}{x!}$ , where x is the observed number and $E_{D}$ is the predicted number ( $E_{pois}$ or $E_{TC}$ ) of three-point alignments.

#### 2.2.6. Comparison of observed versus predicted numbers of conga lines

To calculate the probability of seeing the observed number of conga lines found on each surface by random chance, the following steps were taken:

1. The conga-line-finding algorithm was applied to the point maps of each fossil surface for a range of radius r between 5 and 50 mm, at 1 mm increments, to find the observed number of conga lines on the surface.
2. CSR and TC models were fitted to the surface point pattern, following §2.2.2 (Steps 1 and 2).
3. Model fit was evaluated using Diggle’s goodness-of-fit test and the best-fit model was found.
4. Using the best-fit background distribution, the predicted number of congas for that surface was calculated using equations (2.1) and (2.2) for the range of r .
5. The probability of the observed number of congas found (Step 1) given the predicted number of congas (Step 4), for each value of r , was calculated using the Poisson distribution (§2.2.4).
6. The range of r in which the probability was less than 0.05 was noted, in addition to whether more or fewer congas were found than predicted.

### 2.3. Computational fluid dynamics

Computational fluid dynamics (CFD) is a tool for simulating fluid flow under varying conditions by solving a suite of governing equations that describe the flow. CFD analyses are increasingly used in Ediacaran research to describe how fluids interact with the morphological characteristics of taxa, such as *Tribrachidium* [52], *Parvancorina* [53], *Ernietta* [54,55], *Arkarua* [56]*, Pteridinium* [57] and *Pectinifrons* [58], permitting inference of feeding mode and mobility.

Here, we use established CFD methods alongside discrete phase modelling (DPM). DPM is a particle tracking method that enables the dispersal and settlement of propagules to be modelled [59,60], to test whether the conga lines on the H5 surface could be formed from passive propagule settling in the lee of an existing frond.

#### 2.3.1. Steady-state flow fields

CFD was implemented within Ansys Fluent (2021 R2). The frond was modelled as a rectangle in the centre of a 2 m × 1 m, two-dimensional computational domain, which was large enough to accommodate the extent of downstream eddies and permit the development of a boundary layer [61]. The floor and obstacle were defined by no-slip boundaries, the top of the domain was defined by a free-slip boundary, and the inlet and outlet were defined at opposing ends of the domain. The inlet had a uniform flow profile and the outlet had a pressure equal to the inlet. A quadrilateral dominant mesh with a maximum element size of 5 mm (89 711 elements) was used. Inflation layers, consisting of 24 maximum layers and a growth rate of 1.2, were used to resolve the boundary layer close to the wall, generating a velocity profile consistent with the law of the wall (electronic supplementary material, figure S3). A mesh sensitivity analysis, where the element size was varied between 2 and 10 mm (524 080 to 24 938 elements), was carried out to determine the impact of the mesh parameters on eddy length. A characteristic length of 1 m, at an inlet velocity of 10 cm s^−1^ yielded a Reynold’s number of 65 478, indicative of a turbulent flow regime. Steady-state RANS simulations were run primarily using the k-ω SST turbulence model. The Reynold's stress model is more accurate but does not converge for many target velocities, thus the k–ω SST was used to balance the greatest accuracy with computational tractability (e.g. [55]). Additional parameters used to describe the flow and explanations can be found in electronic supplementary material, §2.3 and tables S4, S5.

The following simulations were carried out:

1. Two-dimensional CFD simulations using the k–ω SST turbulence model were replicated over:
  - A range of frond heights (1, 2, 4, 6, 8 and 10 cm), corresponding to the size distribution of frondose specimens on the H5 surface.
  - A range of velocities (1, 5, 10 and 15 cm s^−1^), that encompass and exceed the range of velocities typical in modern deep-water settings (1–10 cm s^−1^ [62]).
2. A three-dimensional simulation (10 cm s^−1^, 5 × 0.5 × 2 cm frond), which allows features such as horseshoe vortices to be modelled, was run to verify the two-dimensional result.
3. Simulations using the RSM turbulence model were run using a frond height of 5 cm and velocities of 1, 5 and 15 cm s^−1^ to compare to the k–ω SST turbulence model.

#### 2.3.2. Discrete phase modelling

To model the distributions of passive propagules in the simulated flow discrete phase modelling (DPM), a Lagrangian particle tracking technique, was used within Ansys Fluent (2021 R2). The discrete phase (here the propagule particle, parameters found in electronic supplementary material, table S6) is tracked as it interacts with the continuous fluid phase. Here, an uncoupled approach was employed, where the discrete phase does not affect the continuous phase. Steady-state particle tracking was implemented, where the solution of the continuous phase flow was used to calculate the trajectories of the particle. A specified length scale determines the distance a particle travels between trajectory updates, acting as a pseudo-time step. A surface particle injection was defined at the inlet with a flow rate of 1 × 10^−20^ m^−1^. To account for the turbulent dispersion of particles, the discrete random walk model was used with random eddy lifetime (electronic supplementary material, §2.3 and table S6).

When a particle hits the floor, it will settle if the local shear stress is below a critical value ( $\tau_{crit}$ ), otherwise, it will be reflected. This condition was specified using a custom function (https://github.com/kmdelahooke/DPM), where $\tau_{crit}$ = 0.1 Pa, as has been found experimentally for weakly adherent larvae [63]. If a particle settles, its position and velocity are recorded. This information is used to assess how the probability of passive pelagic propagule settlement changes along the *x*-axis in each simulation. DPM simulations were replicated for the range of the frond heights (1, 2, 4, 6, 8 and 10 cm) and velocities (1, 5, 10 and 15 cm s^−1^) used in the CFD simulations, as well as for a range of particle sizes (50 μm, 200 μm, 500 μm, 1 mm and 5 mm), encompassing the range of sizes of extant parenchymella (demosponge) and planula (cnidarian) larvae [64,65].

## 3. Results

### 3.1. Were conga lines formed by chance?

#### 3.1.1. Comparison of analytical and numerical results

The relationship of the predicted number of three-point alignments given a CSR background distribution ( $E_{pois}$ ) with the spacing ( r ) and angle ( θ ) between three-point alignments and the density of the point pattern ( λ ), as well as the relationship of the predicted number of three-point alignments given a TC background distribution ( $E_{TC}$ ) with r , θ and TC parameters κ (density of the parent points), μ (mean points per cluster) and σ (standard deviation of distance of an offspring point to its parent), as determined by both analytical and numeric methods, was compared between these methods (electronic supplementary material, figures S4 and S5). The analytical and numerical data are consistent with one another, with slight discrepancies likely due to the stochastic nature of numerical methods and possibly unaccounted for edge effects [66,67], indicating the validity of analytical methods (electronic supplementary material, figures S4 and S5).

#### 3.1.2. Conga lines—are they real and where are they found?

**Figure 5.**
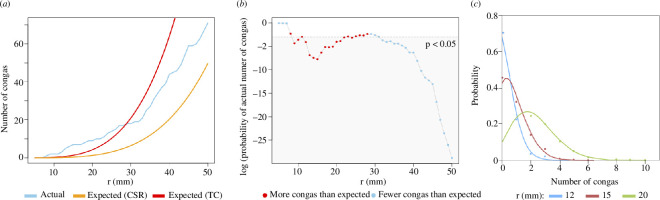
(*a*) Observed (actual) versus predicted (expected) number of conga lines on the H5 fossil surface ( θ = 30). Input data have been retrodeformed. (*b*) Probability of the actual number of conga lines with fronds spaced at a given radius (r) on the H5 surface (retrodeformed), with a Thomas Cluster background distribution. (*c*) Probability distribution of the number of conga lines on the H5 surface for a range of radius spacings. Points result from Monte–Carlo simulations; lines follow a Poisson distribution.

**Figure 6.**
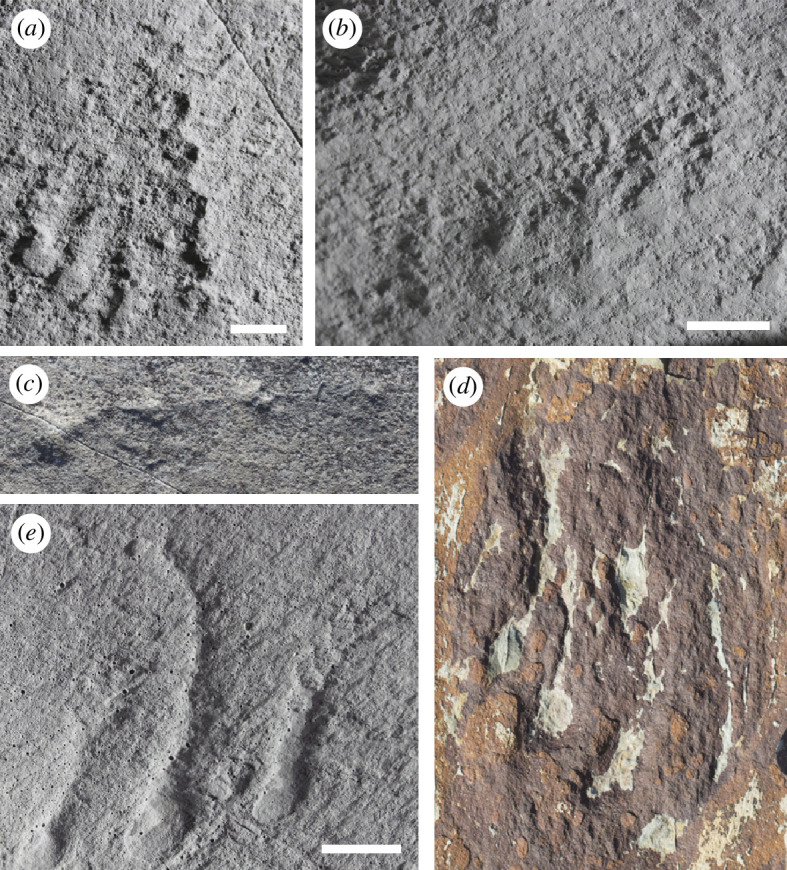
‘Conga lines’ of aligned frondose organisms on late Ediacaran fossil-bearing surfaces from Newfoundland, Canada. (*a*, *b*) H5, Bonavista Peninsula. (*c*) Clapham’s Pigeon Cove, MPER, with a faint linear structure seemingly present connecting the raised circular impressions. (*d*) Effaced specimens on the Brasier/BR5 surface, MPER. (*e*) MUN surface, Bonavista Peninsula. Photographs in (*a*), (*b*) and (*e*) were taken of replica casts housed in the Sedgwick Museum of Earth Sciences, University of Cambridge, UK. Scale bars = 1 cm.

Of the mapped areas, four surfaces have significantly more three-point alignments than are predicted by random chance (H5, MUN, Brasier, CPC), while nine do not (table 1; figure 5; electronic supplementary material, figures S6 and S7; n.b. additional apparent conga lines were observed outside the mapped area on the LMP surface, electronic supplementary material, figure S8). A large number of observed conga lines are composed of effaced (i.e. lacking well-preserved morphological features necessary for taxonomic assignment) fronds on MUN, H5 and Brasier, which comprise a high proportion of overall fossils on each of these surfaces (electronic supplementary material, table S1). The best-preserved conga lines on MUN and H5 both comprise *Charnia* spp. (figure 6*a,b,e*), while on the CPC surface, conga lines are only expressed as linear arrangements of circular raised structures inferred as holdfast discs. One of these conga lines contains a faint filamentous structure running through four of these features (figure 6*c*). These physical observations and probabilistic approaches together indicate that the conga lines likely reflect reproductive processes and are unlikely to have arisen by chance alone, and that they appear to reflect original features of these fossilized bedding plane assemblages.

**Table 1.** Range of distances (r) for which significantly more or fewer three-point alignments (conga lines) are found than predicted.

surface	more congas than predicted - *r* (mm)	fewer congas than predicted *- r* (mm)
Brasier	7–50	N/A
CPC	14–50	N/A
H5 (no retrodeformation)	9–22	33–50
H5 (no retrodeformation, masked)	9–21	32–50
H5 (masked)	9–21	33–50
H5	9–22	N/A
MUN	13–50	N/A

### 3.2. Were conga lines formed by waterborne propagule settling?

#### 3.2.1. Computational fluid dynamics

**Figure 7.**
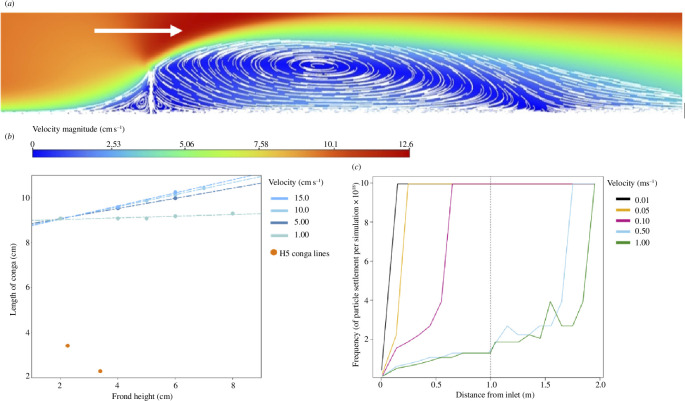
Results of the CFD and DPM analyses. (*a*) Contour plot of velocity magnitude in the lee of an obstacle (white vertical line). Free stream velocity is 10 cm s^–1^, the obstacle is at 1 m and is 5 cm × 0.5 cm in height and width respectively. (*b*) Length of region of lowest velocity for a given frond height and flow velocity (blue), as compared to the observed length of H5 conga lines (orange). (*c*) Frequency of particle settlement along the floor under different velocity regimes. Obstacle is at 1 m. The frequency of settlement only increases after the obstacle when velocity is greater than 0.5 m s^-1^.

Contour plots of flow parameters show that velocity, pressure and wall shear stress drop behind the obstacle (see also electronic supplementary material, figure S9). Two small eddies form either side of the frond, as well as a larger one in the lee (figure 7*a*). These eddies can also be seen as negative stream-wise velocity just above the floor (electronic supplementary material, figure S10). Increasing the height of the obstacle/frond increases the size of the larger lee-side eddy, following a linear relationship (adjusted *R* ^2^ = 0.999, *p* = 0.0002, *F*_1,2_ = 5130; electronic supplementary material, figure S11), thus larger fronds would lead to a larger area of propagule settlement behind them, with longer predicted conga lines. Increasing the velocity also increases the length of the eddy, but nonlinearly, again increasing the predicted length of conga lines. The length of the region of lowest velocity (which is inversely proportional to the probability of propagule settlement) is always much larger than the observed length of Ediacaran conga lines, across frond height and a range of plausible velocities (figure 7*b*). At finer mesh sizes, the length of this zone increases (electronic supplementary material, figure S12), therefore increasing support for this conclusion. When comparing the CFD outputs from different turbulence models, the k–ω SST model (used here) predicts a slightly longer eddy, and thus an even longer conga line behind the frond than the RSM model (electronic supplementary material, figures S13 and S14). Therefore, CFD analyses suggest that for a frond of height <10 cm, the length of the observed conga lines (<4 cm) is much smaller than would be predicted (>8 cm) across all examined flow velocities (figure 7*b*), suggesting that passive pelagic propagule settlement is unlikely to be their formative process.

#### 3.2.2. Discrete phase modelling

Discrete phase modelling (DPM) was used to determine the frequency of particle settlement under different velocity conditions relative to the position of the frond. At low inlet velocities (<10 cm s^−1^), the shear stress is always less than the critical shear velocity (0.1 Pa), thus the frequency of larval settlement is equally high both in front of and behind the frond (figure 7*c*). At high inlet velocities (>50 cm s^−1^), the shear stress is greater than the critical shear stress, except behind the frond. Consequently, the anticipated frequency of propagule settlement increases behind the frond (frequency of propagule settlement is four times higher ~0.5 m behind the frond compared with immediately before; see figure 7*c*). Under conditions typical of the inferred deep-marine setting of H5 [37,68], the flow velocities are considered to have been slow (i.e. <10 cm s^−1^ [62,69]) and hence shear stresses would have been too low to ever re-suspend or prevent the settlement of propagules. When DPM was repeated with different particle sizes, only the smallest particle size (500 μm diameter), showed preferential settling in the lee of the frond (electronic supplementary material, figure S15). Thus, the presence of an obstacle provides no critical benefit to propagule settlement.

## 4. Discussion

Our probabilistic models have shown that the conga lines observed on four late Ediacaran fossil-bearing surfaces in Newfoundland were not the result of chance alignments (table 1; figure 6). CFD and DPM outputs show the region of lowest velocity in the lee of the frond is not comparable to the length of the conga lines observed on H5, such that propagule settlement is not expected to increase in the lee of a frond under low-velocity environmental conditions (<10 cm s^−1^; figure 7*c*). However, it should be noted that the steady-state analyses utilized herein do not consider the impact of fine-scale, ephemeral turbulent structures on propagule settlement. The steady-state results are consistent with the observation that analogous modern features, such as sedimentary obstacle marks [62,70], and preferential larval settlement behind kelp or coral [71,72], are restricted to higher energy environments such as wave-dominated coastal regions and flood events, which provide a sufficient gradient in local shear stresses and velocities to cause preferential deposition behind an obstacle [63,70]. The hypothesis that the observed conga lines are the result of differential passive pelagic propagule settlement around an obstacle can therefore be rejected. Consequently, conga lines are likely indicative of a dispersal-limited reproductive strategy not previously described in rangeomorphs: either through the interaction between philopatric propagules with a current, or a runner-like stolon.

Philopatric propagules are prevalent across many groups of sessile marine invertebrates, including poriferans and cnidarians [42,43,73–76]. In the presence of a current, it is feasible that there could be preferential lee-side recruitment of the propagule relative to its parent, thereby producing a linear feature. Indeed, many conga lines have an axis orthogonal to the felling direction of the fronds (figure 6), perhaps suggesting the influence of a contour current. However, physical evidence for rangeomorph production of buds or fragments is limited [27,30 compare 11], and the preservation of larvae has not been reported in Avalonian strata.

Alternatively, the conga lines could be the consequence of a runner-like stolon, such as those found in hydrozoans [77], anthozoans [78] and entoprocts [79], as well as in algae [80] and terrestrial plants [81]. Stoloniferous reproduction has previously been described in Avalonian communities [11,27], but not in a closely spaced runner-like form. The patterning of conga lines is highly directional, in contrast to the isotropic, network-like pattern of stolons inferred for *Fractofusus* [11]. Similarly, the filamentous connections previously observed between frondose taxa [27] lack the directionality and the close spacing of the conga lines described here. Such variability in stolon patterning mirrors the variability seen in extant stoloniferous metazoans, whose stoloniferous connections are often described on a spectrum between ‘runner-like’ (larger spacing between zooids and rare stolon branching) and ‘sheet-like’ (closely packed zooids and extensive stolon branching) morphs [82]. Ontogeny can control this stolon patterning, e.g. *Hydractinia* colonies transition from more runner-like to more sheet-like as they develop [83]. Runner-like forms are also typically associated with nutrient-poor, resource heterogeneous and disturbed modern environments [80,84,85]. For example, the soft coral *Efflatounaria* shows rapid stolon extension, creating a more runner-like form, as a plastic response to environmental disturbance [84]. Such stolon forms, with minimal branching and spaced-out zooids, enable efficient colonization of the bare substrate as well as escape from poor environmental conditions, a ‘guerrilla’ strategy that is akin to an optimal foraging strategy but for sessile organisms [86,87]. By contrast, sheet-like forms, with extensive branching and close-spaced zooids, reflect a ‘phalanx’ strategy, allowing organisms to capitalize on amenable conditions in their local vicinity [86].

The close-spacing of individual fronds in the conga lines is at odds with the adaptive benefit of a ‘guerrilla’ strategy for runner-like stolon, however, this may suggest that they may reflect an alternative strategy, or indeed no strategy at all. Furthermore, a runner-like stolon is not preserved in any conga lines bearing fronds detected in this study, with the exception of the filamentous structure running through inferred holdfast structures on Clapham’s Pigeon Cove surface (figure 6*c*). This lack of preservation is also the case for inferred *Fractofusus* stolons [11] and may reflect either a taphonomic absence or a transient existence of the original biological stolon structure. In contrast to most modern benthos, the Ediacaran seafloor was often covered by a microbial mat, preserved as disparate textures on bedding planes [88,89]. A mat provides a possible explanation for a preservational bias: if the stolon was embedded in the mat (perhaps for stability) or overgrown by later mat growth, then it may lie beneath the plane of preservation [27]. Alternatively, the stolon could be ephemeral and no longer be present at the time the surface was preserved. Although most extant metazoan stolons are retained throughout the lifespan of the colony and provide a means of nutrient transfer, some stolons may disappear after a daughter colony is established (e.g. [90,91]). The adaptive benefit of a runner-like stolon may differ from modern analogues, but the Ediacaran conga lines are consistent with a stoloniferous formative mechanism.

Conga lines represent an additional strategy of rangeomorph reproduction, augmenting previously described propagules [11,26,28], putative buds and fragments [29,30] and previously described forms of stolons [11,27]. These different reproductive modes may be solely associated with specific taxa, or may instead reflect mixed reproductive strategies within individual taxa capable of employing multiple modes simultaneously [3], reproductive plasticity [1,2] or a combination of both options. On the basis of available evidence, conga lines do not appear to represent a reproductive mode that defines a single taxon. There is no clear signal regarding the constituent taxa of conga lines because a large proportion of specimens are effaced (electronic supplementary material, table S1), but both the MUN and H5 surfaces contain seemingly monospecific conga lines formed of *Charnia* spp. (figure 6*a,b,e*). However, not all *Charnia* or indeed effaced frond specimens on a given surface are associated with conga lines. *Charnia* has been shown to form small clusters on Bed B, Charnwood, UK [25], and some *Charnia* specimens are associated with long filamentous structures on bed LC6 in Newfoundland [27], demonstrating that some of the taxa forming conga lines were also capable of other reproductive strategies. Based on this evidence, we cannot yet determine whether conga lines reflect reproductive plasticity or are one of multiple simultaneous reproductive modes employed by individual organisms.

Mixed reproductive strategies, whether plastic or simultaneous, have an adaptive benefit in variable environments. Plastic strategies conditional on environmental cues produce, in theory, an optimal phenotype for the conditions [92], and are adaptive when the timescale of environmental variability is greater than the lifetime of the organism [93]. In contrast, stochastically mediated plasticity or multiple synchronous reproductive modes can be viewed as bet-hedging strategies [92]. Such strategies produce a suboptimal phenotype, with lower instantaneous fitness but greater average fitness, thereby creating an evolutionary stable strategy. Modelling of multiple reproductive strategies in a fluctuating environment has found that the coexistence of two or more reproductive modes is favoured in all situations except when the local extinction risk is at the extremes (i.e. within variable environments), and thus multiple strategies can be viewed as indicative of variable environments [80]. Therefore, mixed rangeomorph reproductive strategies may reflect an adaption to environmental variability, which could be spatial or temporal.

Limited data exist regarding spatial environmental variability within late Ediacaran deep-marine depositional environments. The sedimentology of bedding planes in instances where a fossiliferous bedding plane outcrops multiple times over several kilometres is largely invariant [94]. Small-scale spatial environmental heterogeneity may exist, such as where a microbial mat or a local nutrient source is present [88,89], though previous work has shown a limited impact of inferred small-scale heterogeneities on community dynamics [25,33]. Temporal environmental variation may have resulted from episodic disturbances by the turbidites and ash falls that characterize the succession [9,95,96] or from varying nutrient levels, temperatures or redox conditions, for which there is currently little bed-scale information from Avalonian settings. Although the frequency of disturbance from ash or turbidite events is not known, modern analogues have a periodicity of 10–200 years [97,98], which are likely to typically exceed rangeomorph lifespans. As such, it is plausible that temporal environmental variability may be a driver of mixed reproductive strategies, in particular reproductive plasticity, in rangeomorph populations.

Using mixed reproductive strategies would have enabled rangeomorphs to act as reproductive generalists under multiple conditions of environmental variation, allowing them to colonize large areas of the seafloor and achieve a global palaeogeographic distribution [11,28,99], as well as withstand environmental perturbations [96]. Such reproductive strategies perhaps contributed to their persistence in late Ediacaran ecosystems for tens of millions of years [9,100]. The use of mixed reproductive strategies by rangeomorphs demonstrates the ancient origins of this strategy, which characterizes the success of many modern marine invertebrate groups [4] and may have played a role in facilitating the initial success and global expansion of early macroscopic metazoans.

## 5. Conclusion

We describe novel ‘conga lines’, alignments of closely spaced frondose macro-organisms, from four bedding plane surfaces in the late Ediacaran of Newfoundland. Using probabilistic modelling, we show that these features did not form by random chance. Computational fluid dynamics, in particular discrete phase modelling, demonstrate that they did not result from passive pelagic propagule settling. We thus conclude that the conga lines were most likely formed by closely spaced, runner-like stolons, though current-influenced philopatric propagules cannot be entirely dismissed. Conga lines provide a new line of evidence in support of mixed rangeomorph reproductive strategies, in particular dispersal limitation associated with clonal organisms. Such strategies reflect evolutionary trade-offs in the earliest animal communities, perhaps in response to environmental instability, and may have contributed to the persistence and dominance of rangeomorphs in certain late Ediacaran marine settings.

## Data Availability

Data can be found at https://doi.org/10.6084/m9.figshare.25314391 and https://doi.org/10.6084/m9.figshare.24270499. Electronic supplementary material is available online [101].

## References

[1] Reitzel AM , Stefanik D , Finnerty JR . 2011 Asexual reproduction in Cnidaria: comparative developmental processes and candidate mechanisms. In Mechanisms of life history evolution. The genetics and physiology of life history traits and trade–offs, pp. 101–113. Oxford: Oxford University Press. (10.1093/acprof:oso/9780199568765.001.0001)

[2] Ryan WH , Miller TE . 2019 Reproductive strategy changes across latitude in a clonal sea anemone. Mar. Ecol. Prog. Ser. **611** , 129–141. (10.3354/meps12862)

[3] Bastidas C , Fabricius KE , Willis BL . 2004 Demographic aspects of the soft coral *Sinularia flexibilis* leading to local dominance on coral reefs. In Coelenterate biology 2003: trends in research on Cnidaria and Ctenophora, pp. 433–441. New York: Springer. (10.1007/978-1-4020-2762-8)

[4] D’Ambra I , Merquiol L , Graham WM , Costello JH . 2021 “Indirect development” increases reproductive plasticity and contributes to the success of scyphozoan jellyfish in the oceans. Sci. Rep. **11** , 18653. (10.1038/s41598-021-98171-w) 34545165 PMC8452738

[5] Xiao SH , Narbonne GM . 2020 The Ediacaran Period. In Geologic time scale, pp. 521–561. Elsevier B.V. (10.1016/B978-0-12-824360-2.00018-8)

[6] Liu AG , Kenchington CG , Mitchell EG . 2015 Remarkable insights into the paleoecology of the Avalonian Ediacaran macrobiota. Gondwana Res. **27** , 1355–1380. (10.1016/j.gr.2014.11.002)

[7] Noble SR , Condon DJ , Carney JN , Wilby PR , Pharaoh TC , Ford TD . 2015 U-Pb geochronology and global context of the Charnian Supergroup, UK: Constraints on the age of key Ediacaran fossil assemblages. Geol. Soc. Am. Bull. **127** , 250–265. (10.1130/B31013.1)

[8] Boag TH , Darroch SAF , Laflamme M . 2016 Ediacaran distributions in space and time: testing assemblage concepts of earliest macroscopic body fossils. Paleobiology **42** , 574–594. (10.1017/pab.2016.20)

[9] Matthews JJ , Liu AG , Yang C , McIlroy D , Levell B , Condon DJ . 2021 A chronostratigraphic framework for the rise of the Ediacaran macrobiota: new constraints from Mistaken Point Ecological Reserve, Newfoundland. GSA Bul. **133** , 612–624. (10.1130/B35646.1)

[10] Waggoner B . 2003 The Ediacaran biotas in space and time. Integr. Comp. Biol. **43** , 104–113. (10.1093/icb/43.1.104) 21680415

[11] Mitchell EG , Kenchington CG , Liu AG , Matthews JJ , Butterfield NJ . 2015 Reconstructing the reproductive mode of an Ediacaran macro-organism. Nature **524** , 343–346. (10.1038/nature14646) 26237408

[12] Wood DA , Dalrymple RW , Narbonne GM , Gehling JG , Clapham ME . 2003 Paleoenvironmental analysis of the late Neoproterozoic Mistaken Point and Trepassey formations, Southeastern Newfoundland. Can. J. Earth Sci. **40** , 1375–1391. (10.1139/e03-048)

[13] O’Brien SJ , King AF . 2005 Late Neoproterozoic (Ediacaran) stratigraphy of Avalon zone sedimentary rocks, Bonavista Peninsula, Newfoundland. Curr. Res. Nfld. Labrador Dep. Nat. Resour. Geol. Surv **5** , 101–113.

[14] Ichaso AA , Dalrymple RW , Narbonne GM . 2007 Paleoenvironmental and basin analysis of the late Neoproterozoic (Ediacaran) Upper Conception and St. John’s groups, West Conception Bay, Newfoundland. Can. J. Earth Sci. **44** , 25–41. (10.1139/e06-098)

[15] Narbonne GM . 2004 Modular construction of early Ediacaran complex life forms. Science **305** , 1141–1144. (10.1126/science.1099727) 15256615

[16] Laflamme M , Darroch SAF , Tweedt SM , Peterson KJ , Erwin DH . 2013 The end of the Ediacara biota: extinction, biotic replacement, or Cheshire Cat? Gondwana Res. **23** , 558–573. (10.1016/j.gr.2012.11.004)

[17] Hoyal Cuthill JF . 2022 Ediacaran survivors in the Cambrian: suspicions, denials and a smoking gun. Geol. Mag. **159** , 1210–1219. (10.1017/S0016756821001333)

[18] Hu S , Zhao F , Liu AG , Zhu M . 2023 A new Cambrian frondose organism: ‘Ediacaran survivor’ or convergent evolution? J. Geol. Soc. Lond. **180** , jgs2022–088. (10.1144/jgs2022-088)

[19] Dunn FS , Liu AG , Grazhdankin DV , Vixseboxse P , Flannery-Sutherland J , Green E , Harris S , Wilby PR , Donoghue PCJ . 2021 The developmental biology of Charnia and the eumetazoan affinity of the Ediacaran rangeomorphs. Sci. Adv. **7** , eabe0291. (10.1126/sciadv.abe0291) 34301594 PMC8302126

[20] Dunn FS , Liu AG , Gehling JG . 2019 Anatomical and ontogenetic reassessment of the Ediacaran frond Arborea arborea and its placement within total group Eumetazoa. Palaeontology **62** , 851–865. (10.1111/pala.12431)

[21] Dunn FS , Kenchington CG , Parry LA , Clark JW , Kendall RS , Wilby PR . 2022 A crown-group cnidarian from the Ediacaran of Charnwood Forest, UK. Nat. Ecol. Evol. **6** , 1095–1104. (10.1038/s41559-022-01807-x) 35879540 PMC9349040

[22] Liu AG , Matthews JJ , Menon LR , McIlroy D , Brasier MD . 2014 Haootia quadriformis n. gen., n. sp., interpreted as a muscular cnidarian impression from the late Ediacaran period (approx. 560 Ma). Proc. R. Soc. B Biol. Sci. **281** , 20141202. (10.1098/rspb.2014.1202) PMC4173675 25165764

[23] Sperling EA , Peterson KJ , Laflamme M . 2011 Rangeomorphs, Thectardis (Porifera?) and dissolved organic carbon in the Ediacaran oceans. Geobiology **9** , 24–33. (10.1111/j.1472-4669.2010.00259.x) 21044251

[24] Suarez PA , Leys SP . 2022 The sponge pump as a morphological character in the fossil record. Paleobiology **48** , 446–461. (10.1017/pab.2021.43)

[25] Mitchell EG , Harris S , Kenchington CG , Vixseboxse P , Roberts L , Clark C , Dennis A , Liu AG , Wilby PR . 2019 The importance of neutral over niche processes in structuring Ediacaran early animal communities. Ecol. Lett. **22** , 2028–2038. (10.1111/ele.13383) 31515929 PMC6899650

[26] Clapham ME , Narbonne GM , Gehling JG . 2003 Paleoecology of the oldest known animal communities: Ediacaran assemblages at Mistaken Point, Newfoundland. Paleobiology **29** , 527–544. (10.1666/0094-8373(2003)029<0527:POTOKA>2.0.CO;2)

[27] Liu AG , Dunn FS . 2020 Filamentous connections between Ediacaran fronds. Curr. Biol. **30** , 1322–1328. (10.1016/j.cub.2020.01.052) 32142705

[28] Darroch SAF , Laflamme M , Clapham ME . 2013 Population structure of the oldest known macroscopic communities from Mistaken Point, Newfoundland. Paleobiology **39** , 591–608. (10.1666/12051)

[29] Narbonne GM , Laflamme M , Greentree C , Trusler P . 2009 Reconstructing a lost world: Ediacaran rangeomorphs from Spaniard’s Bay, Newfoundland. J. Paleontol. **83** , 503–523. (10.1666/08-072R1.1)

[30] Pasinetti G , McIlroy D . 2023 Palaeobiology and taphonomy of the rangeomorph Culmofrons plumosa. Palaeontology **66** , e12671. (10.1111/pala.12671)

[31] Gori A , Rossi S , Berganzo E , Pretus JL , Dale MRT , Gili JM . 2011 Spatial distribution patterns of the gorgonians Eunicella singularis, Paramuricea clavata, and Leptogorgia sarmentosa (Cape of Creus, Northwestern Mediterranean Sea). Mar. Biol **158** , 143–158. (10.1007/s00227-010-1548-8)

[32] Prado E , Sánchez F , Rodríguez-Basalo A , Altuna Á , Cobo A . 2019 Analysis of the population structure of a gorgonian forest (Placogorgia sp.) using a photogrammetric 3D modeling approach at Le Danois Bank, Cantabrian Sea. Deep Sea Res. Part Oceanogr. Res. Pap. **153** , 103124. (10.1016/j.dsr.2019.103124)

[33] Mitchell EG , Butterfield NJ . 2018 Spatial analyses of Ediacaran communities at Mistaken Point. Paleobiology **44** , 40–57. (10.1017/pab.2017.35)

[34] Mitchell EG , Kenchington CG . 2018 The utility of height for the Ediacaran organisms of Mistaken Point. Nat. Ecol. Evol. **2** , 1218–1222. (10.1038/s41559-018-0591-6) 29942022

[35] Wang J *et al* . 2020 Evolutionary transcriptomics of metazoan biphasic life cycle supports a single intercalation origin of metazoan larvae. Nat. Ecol. Evol. **4** , 725–736. (10.1038/s41559-020-1138-1) 32203475

[36] Hoyal Cuthill JF , Conway Morris S . 2014 Fractal branching organizations of Ediacaran rangeomorph fronds reveal a lost Proterozoic body plan. Proc. Natl Acad. Sci. USA **111** , 13122–13126. (10.1073/pnas.1408542111) 25114255 PMC4246981

[37] Hofmann HJ , O’Brien SJ , King AF . 2008 Ediacaran biota on Bonavista Peninsula, Newfoundland, Canada. J. Paleontol. **82** , 1–36. (10.1666/06-087.1)

[38] Wiegand T , Moloney KA . 2013 Handbook of Spatial Point-Pattern Analysis in Ecology. Boca Raton, FL: CRC Press. (10.1201/b16195)

[39] Havenhand JN . 1995 Evolutionary Ecology of larval types. In Ecology of Marine Invertebrate larvae, pp. 79–122. Boca Raton, FL: CRC Press (CRC Marine Science Series).

[40] McEdward L . 1995 Ecology of marine invertebrate larvae. Boca Raton, FL: CRC Press (CRC Marine Science Series).

[41] Dahan M , Benayahu Y . 1997 Clonal propagation by the azooxanthellate octocoral Dendronephthya hemprichi *.* Coral Reefs **16** , 5–12. (10.1007/s003380050053)

[42] Shanks AL . 2009 Pelagic larval duration and dispersal distance revisited. Biol. Bull. **216** , 373–385. (10.1086/BBLv216n3p373) 19556601

[43] Waller RG , Goode S , Tracey D , Johnstone J , Mercier A . 2023 A review of current knowledge on reproductive and larval processes of deep-sea corals. Mar. Biol. **170** , 58. (10.1007/s00227-023-04182-8)

[44] Stephenson NP , Delahooke KM , Barnes N , Rideout BWT , Kenchington CG , Manica A , Mitchell EG . 2024 Morphology shapes community dynamics in early animal ecosystems. Nat. Ecol. Evol (10.1038/s41559-024-02422-8) PMC11239517 38867093

[45] Liu AG , Matthews JJ , McIlroy D . 2016 The Beothukis/Culmofrons problem and its bearing on Ediacaran macrofossil taxonomy: evidence from an exceptional new fossil locality. Palaeontology **59** , 45–58. (10.1111/pala.12206)

[46] Baddeley A , Turner R . 2005 Spatstat: an R package for analyzing spatial point patterns. J. Stat. Softw **12** , 1–42. (10.18637/jss.v012.i06)

[47] Illian J , Penttinen A , Stoyan H , Stoyan D . 2007 Statistical analysis and modelling of spatial point patterns. Hoboken: John Wiley & Sons.

[48] Baddeley A , Turner R . 2000 Practical maximum pseudolikelihood for spatial point patterns. Aust. NZ J. Stat. **42** , 283–322. (10.1111/1467-842X.00128)

[49] Diggle PJ , Gratton RJ . 1984 Monte Carlo methods of inference for implicit statistical models. J. R. Stat. Soc. Ser. B Methodol. **46** , 193–212. (10.1111/j.2517-6161.1984.tb01290.x)

[50] Diggle P . 2003 Statistical analysis of spatial point patterns, 2nd edn. London, New York: Arnold: Oxford University Press.

[51] Baddeley A , Rubak E , Turner R . 2015 Spatial point patterns: methodology and applications with R. Boca Raton, FL: CRC Press.

[52] Rahman IA , Darroch SAF , Racicot RA , Laflamme M . 2015 Suspension feeding in the enigmatic Ediacaran organism Tribrachidium demonstrates complexity of Neoproterozoic ecosystems. Sci. Adv. **1** , e1500800. (10.1126/sciadv.1500800) 26702439 PMC4681345

[53] Darroch SAF , Rahman IA , Gibson B , Racicot RA , Laflamme M . 2017 Inference of facultative mobility in the enigmatic Ediacaran organism Parvancorina. Biol. Lett. **13** , 20170033. (10.1098/rsbl.2017.0033) 28515329 PMC5454237

[54] Gibson BM , Rahman IA , Maloney KM , Racicot RA , Mocke H , Laflamme M , Darroch SAF . 2019 Gregarious suspension feeding in a modular Ediacaran organism. Sci. Adv. **5** , eaaw0260. (10.1126/sciadv.aaw0260) 31223655 PMC6584682

[55] Gibson BM , Furbish DJ , Rahman IA , Schmeeckle MW , Laflamme M , Darroch SAF . 2021 Ancient life and moving fluids. Biol. Rev. Camb. Philos. Soc. **96** , 129–152. (10.1111/brv.12649) 32959981 PMC7821342

[56] Cracknell K , García-Bellido DC , Gehling JG , Ankor MJ , Darroch SAF , Rahman IA . 2021 Pentaradial eukaryote suggests expansion of suspension feeding in White Sea-aged Ediacaran communities. Sci. Rep. **11** , 4121. (10.1038/s41598-021-83452-1) 33602958 PMC7893023

[57] Darroch SA , et al . 2022 The life and times of Pteridinium simplex. Paleobiology **48** , 527–556. (10.1017/pab.2022.2)

[58] Darroch SAF *et al* . 2023 The rangeomorph Pectinifrons abyssalis: hydrodynamic function at the dawn of animal life. iScience **26** , 105989. (10.1016/j.isci.2023.105989) 36756377 PMC9900436

[59] Zahari NM , Zawawi MH , Sidek LM , Mohamad D , Itam Z , Ramli MZ , Syamsir A , Abas A , Rashid M . 2018 Introduction of discrete phase model (DPM) in fluid flow: A review. In AIP Conference Proceedings. AIP Publishing LLC. (10.1063/1.5066875)

[60] Dynowski JF , Nebelsick JH , Klein A , Roth-Nebelsick A . 2016 Computational fluid dynamics analysis of the fossil crinoid Encrinus liliiformis (Echinodermata: Crinoidea). PLoS One **11** , e0156408. (10.1371/journal.pone.0156408) 27243221 PMC4887110

[61] Thomsen L , Flach E . 1997 Mesocosm observations of fluxes of particulate matter within the benthic boundary layer. J. Sea Res. **37** , 67–79. (10.1016/S1385-1101(97)00004-X)

[62] Stow D , Smillie Z . 2020 Distinguishing between deep-water sediment facies: turbidites, contourites and hemipelagites. Geosciences **10** , 68. (10.3390/geosciences10020068)

[63] Reidenbach MA , Koseff JR , Koehl MAR . 2009 Hydrodynamic forces on larvae affect their settlement on coral reefs in turbulent, wave-driven flow. Limnol. Oceanogr. **54** , 318–330. (10.4319/lo.2009.54.1.0318)

[64] Isomura N , Nishihira M . 2001 Size variation of planulae and its effect on the lifetime of planulae in three pocilloporid corals. Coral Reefs **20** , 309–315. (10.1007/s003380100180)

[65] Maldonado M . 2002 Phylum Porifera. In Atlas of marine invertebrate larvae, pp. 21–50. San Diego: Academic Press.

[66] Ripley BD . 1977 Modelling spatial patterns. J. R. Stat. Soc. Ser. B Methodol. **39** , 172–192. (10.1111/j.2517-6161.1977.tb01615.x)

[67] Goreaud F , Pélissier R . 1999 On explicit formulas of edge effect correction for Ripley’s K ‐function. J. Veg. Sci. **10** , 433–438. (10.2307/3237072)

[68] Mason SJ , Narbonne GM , Dalrymple RW , O’Brien SJ . 2013 Paleoenvironmental analysis of Ediacaran strata in the Catalina Dome, Bonavista Peninsula, Newfoundland. Can. J. Earth Sci. **50** , 197–212. (10.1139/cjes-2012-0099)

[69] Miramontes E , Eggenhuisen JT , Jacinto RS , Poneti G , Pohl F , Normandeau A , Campbell DC , Hernández-Molina FJ . 2020 Channel-levee evolution in combined contour current–turbidity current flows from flume-tank experiments. Geology **48** , 353–357. (10.1130/G47111.1)

[70] Euler T , Herget J . 2012 Controls on local scour and deposition induced by obstacles in fluvial environments. Catena **91** , 35–46. (10.1016/j.catena.2010.11.002)

[71] Koehl MAR , Hadfield MG . 2010 Hydrodynamics of larval settlement from a larva’s point of view. Integr. Comp. Biol. **50** , 539–551. (10.1093/icb/icq101) 21558223

[72] Rosman JH , Koseff JR , Monismith SG , Grover J . 2007 A field investigation into the effects of a kelp forest (Macrocystis pyrifera) on coastal hydrodynamics and transport. J. Geophys. Res. Oceans **112** , C02016. (10.1029/2005JC003430)

[73] Jackson JBC . 1986 Modes of dispersal of clonal benthic invertebrates: consequences for species’ distributions and genetic structure of local populations. Bull. Mar. Sci. **39** , 588–606.

[74] Miller KJ . 1998 Short-distance dispersal of black coral larvae: inference from spatial analysis of colony genotypes. Mar. Ecol. Prog. Ser. **163** , 225–233. (10.3354/meps163225)

[75] Blanquer A , Uriz MJ , Caujapé-Castells J . 2009 Small-scale spatial genetic structure in Scopalina lophyropoda, an encrusting sponge with philopatric larval dispersal and frequent fission and fusion events. Mar. Ecol. Prog. Ser. **380** , 95–102. (10.3354/meps07931)

[76] Winston JE . 2012 Dispersal in marine organisms without a pelagic larval phase. Integr. Comp. Biol. **52** , 447–457. (10.1093/icb/ics040) 22505589

[77] Cartwright P , Travert MK , Sanders SM . 2021 The evolution and development of coloniality in hydrozoans. J. Exp. Zool. B Mol. Dev. Evol. **336** , 293–299. (10.1002/jez.b.22996) 32798274

[78] McFadden CS , van Ofwegen LP . 2012 Stoloniferous octocorals (Anthozoa, Octocorallia) from South Africa, with descriptions of a new family of Alcyonacea, a new genus of Clavulariidae, and a new species of Cornularia (Cornulariidae). Invert. Syst. **26** , 331. (10.1071/IS12035)

[79] Nielsen C . 2005 Entoprocta. In Encyclopedia of life sciences, pp. 345–346, vol. **6** . Hoboken: John Wiley & Sons, Ltd.

[80] Collado-Vides L . 2002 Morphological plasticity of Caulerpa prolifera (Caulerpales, Chlorophyta) in relation to growth form in a coral reef lagoon. Bot. Mar. **45** , 123–129. (10.1515/BOT.2002.013)

[81] Savini G , Giorgi V , Scarano E , Neri D . 2008 Strawberry plant relationship through the stolon. Physiol. Plant. **134** , 421–429. (10.1111/j.1399-3054.2008.01145.x) 18533001

[82] Blackstone NW , Buss LW . 1991 Shape variation in hydractiniid hydroids. Biol. Bull. **180** , 394–405. (10.2307/1542340) 29304663

[83] Cartwright P , Schierwater B , Buss LW . 2006 Expression of a Gsx parahox gene,Cnox-2, in colony ontogeny in Hydractinia (Cnidaria: Hydrozoa). J. Exp. Zool. **306B** , 460–469. (10.1002/jez.b.21106) 16615106

[84] Karlson RH , Hughes TP , Karlson SR . 1996 Density‐dependent dynamics of soft coral aggregations: the significance of clonal growth and form. Ecology **77** , 1592–1599. (10.2307/2265554)

[85] Vogt KSC , Harmata KL , Coulombe HL , Bross LS , Blackstone NW . 2011 Causes and consequences of stolon regression in a colonial hydroid. J. Exp. Biol. **214** , 3197–3205. (10.1242/jeb.057430) 21900467

[86] Clegg LM . 1978 The morphology of clonal growth and its relevance to the population dynamics of perennial plants. PhD Thesis. [UCNW, Bangor: Plant Biology]: University of Wales.

[87] Hutchings MJ . 1988 Differential foraging for resources, and structural plasticity in plants. Trends Ecol. Evol. **3** , 200–204. (10.1016/0169-5347(88)90007-9) 21227201

[88] Gehling JG , Droser ML . 2009 Textured organic surfaces associated with the Ediacara biota in South Australia. Earth-Sci. Rev. **96** , 196–206. (10.1016/j.earscirev.2009.03.002)

[89] Mángano G , Buatois LA . 2017 The Cambrian revolutions: trace-fossil record, timing, links and geobiological impact. Earth-Sci. Rev. **173** , 96–108. (10.1016/j.earscirev.2017.08.009)

[90] Fabricius KE , Alderslade P . 2001 Soft corals and sea fans: a comprehensive guide to the tropical shallow water genera of the Central-West Pacific, the Indian Ocean and the Red Sea. Australian Institute of Marine Science.

[91] Lavrov AI , Kosevich IA . 2014 Sponge cell reaggregation: mechanisms and dynamics of the process. Russ. J. Dev. Biol. **45** , 205–223. (10.1134/S1062360414040067) 25735148

[92] Simons AM . 2011 Modes of response to environmental change and the elusive empirical evidence for bet hedging. Proc. R. Soc. B Biol. Sci. **278** , 1601–1609. (10.1098/rspb.2011.0176) PMC3081777 21411456

[93] Fusco G , Minelli A . 2010 Phenotypic plasticity in development and evolution: facts and concepts. Philos. Trans. R. Soc. Lond. B. Biol. Sci. **365** , 547–556. (10.1098/rstb.2009.0267) 20083631 PMC2817147

[94] Matthews JJ , Liu AG , McIlroy D . 2017 Post-fossilization processes and their implications for understanding Ediacaran macrofossil assemblages. Geol. Soc. London Spec. Publ. **448** , 251–269. (10.1144/SP448.20)

[95] Carney JN . 1999 Revisiting the Charnian Supergroup: new advances in understanding old rocks. Geol. Today **15** , 221–229. (10.1046/j.1365-2451.1999.00006.x)

[96] Wilby PR , Kenchington CG , Wilby RL . 2015 Role of low intensity environmental disturbance in structuring the earliest (Ediacaran) macrobenthic tiered communities. Palaeogeogr. Palaeoclimatol. Palaeoecol. **434** , 14–27. (10.1016/j.palaeo.2015.03.033)

[97] Huh CA , Su CC , Wang CH , Lee SY , Lin IT . 2006 Sedimentation in the Southern Okinawa Trough — rates, turbidites and a sediment budget. Mar. Geol. **231** , 129–139. (10.1016/j.margeo.2006.05.009)

[98] Harris PT . 2014 Shelf and deep-sea sedimentary environments and physical benthic disturbance regimes: a review and synthesis. Mar. Geol. **353** , 169–184. (10.1016/j.margeo.2014.03.023)

[99] Boddy CE , Mitchell EG , Merdith A , Liu AG . 2022 Palaeolatitudinal distribution of the Ediacaran macrobiota. J. Geol. Soc. Lond. **179** , jgs2021–030. (10.1144/jgs2021-030)

[100] Wood R , Liu AG , Bowyer F , Wilby PR , Dunn FS , Kenchington CG , Hoyal Cuthill JF , Mitchell EG , Penny A . 2019 Integrated records of environmental change and evolution challenge the Cambrian explosion. Nat. Ecol. Evol. **3** , 528–538. (10.1038/s41559-019-0821-6) 30858589

[101] Delahooke KM , Liu AG , Stephenson NP , Mitchell EG . 2024 Supplementary material from: Conga lines of Ediacaran fronds: insights into the reproductive biology of early metazoans. Figshare. (10.6084/m9.figshare.c.7214462) PMC11286166 39076788

